# ATP Synthase Deficiency due to TMEM70 Mutation Leads to Ultrastructural Mitochondrial Degeneration and Is Amenable to Treatment

**DOI:** 10.1155/2015/462592

**Published:** 2015-10-13

**Authors:** Anne K. Braczynski, Stefan Vlaho, Klaus Müller, Ilka Wittig, Anna-Eva Blank, Dominique S. Tews, Ulrich Drott, Stephanie Kleinle, Angela Abicht, Rita Horvath, Karl H. Plate, Werner Stenzel, Hans H. Goebel, Andreas Schulze, Patrick N. Harter, Matthias Kieslich, Michel Mittelbronn

**Affiliations:** ^1^Edinger Institute, Institute of Neurology, Goethe University, 60528 Frankfurt am Main, Germany; ^2^Department of Neuropediatrics, Goethe University, 60590 Frankfurt am Main, Germany; ^3^Functional Proteomics, SFB815 Core Unit, Faculty of Medicine, Goethe University, Theodor-Stern-Kai 7, 60590 Frankfurt am Main, Germany; ^4^Medical Genetic Center, 80336 Munich, Germany; ^5^Institute of Genetic Medicine, Newcastle University, Newcastle upon Tyne, Tyne and Wear NE1 3BZ, UK; ^6^Department of Neuropathology, Charité, 10117 Berlin, Germany; ^7^Department of Neuropathology, University Hospital, Johannes Gutenberg University Mainz, 55131 Mainz, Germany; ^8^Division of Clinical and Metabolic Genetics, The Hospital for Sick Children and University of Toronto, Toronto, ON, Canada M5G 1X8; ^9^Genetics and Genome Biology, Peter Gilgan Center for Research and Learning, Toronto, ON, Canada M5G 0A4

## Abstract

TMEM70 is involved in the biogenesis of mitochondrial ATP synthase and mutations in the *TMEM70* gene impair oxidative phosphorylation. Herein, we report on pathology and treatment of ATP synthase deficiency in four siblings. A consanguineous family of Roma (Gipsy) ethnic origin gave birth to 6 children of which 4 were affected presenting with dysmorphic features, failure to thrive, cardiomyopathy, metabolic crises, and 3-methylglutaconic aciduria as clinical symptoms. Genetic testing revealed a homozygous mutation (c.317-2A>G) in the *TMEM70* gene. While light microscopy was unremarkable, ultrastructural investigation of muscle tissue revealed accumulation of swollen degenerated mitochondria with lipid crystalloid inclusions, cristae aggregation, and exocytosis of mitochondrial material. Biochemical analysis of mitochondrial complexes showed an almost complete ATP synthase deficiency. Despite harbouring the same mutation, the clinical outcome in the four siblings was different. Two children died within 60 h after birth; the other two had recurrent life-threatening metabolic crises but were successfully managed with supplementation of anaplerotic amino acids, lipids, and symptomatic treatment during metabolic crisis. In summary, *TMEM70* mutations can cause distinct ultrastructural mitochondrial degeneration and almost complete deficiency of ATP synthase but are still amenable to treatment.

## 1. Introduction

Skeletal muscle function strongly depends on adenosine triphosphate (ATP) providing energy in most energy consuming cellular processes. In mitochondria, aerobic ATP is generated via oxidative phosphorylation (OXPHOS) comprising several respiratory chain subunits of which complexes I-IV create a proton gradient across the inner mitochondrial membrane and complex V, also named ATP synthase, transfers protons back to the inner mitochondrial membrane [1, 2]. The mitochondrial respiratory chain consists of over 90 mainly nuclear-coded proteins. Complex II of OXPHOS is the only complex which is exclusively nuclear-coded. Mitochondrial DNA (mtDNA) consists of 37 genes encoding 13 mitochondrial proteins exerting functions in the OXPHOS system [3]. ATP synthase (complex V) is composed of two functional domains: F_1_ situated in the mitochondrial matrix and F_0_ in the inner mitochondrial membrane [4]. It is assembled of 16 subunits. Two subunits (ATPase 6 and ATPase 8) are encoded by mitochondrial DNA (*MT-ATP6; MT-ATP8*); the remaining 14 subunits of complex V are coded by nuclear DNA. Complex V seems to play an important role in mitochondria morphology [5]. Although being rare, there are several complex V related disorders including mitochondrial and nuclear gene defects. Defects of nuclear genes resulting in complex V deficiency are located in* ATPAF2, ATP5E, ATP5A1*, and* TMEM70* genes. Out of these mutations patients with transmembrane protein 70 (*TMEM70)* mutations most often present with neonatal onset of elevated plasma lactate levels, 3-methylglutaconic aciduria (3-MGA), cardiomyopathy, facial dysmorphism, and psychomotor and mental retardation [6, 7] and emerged to be clinically well characterized since the gene description in 2008. The* TMEM70* gene on chromosome 8q21.11 contains 3 exons which code for this 21 kD protein, which is probably located in the inner mitochondrial membrane [8]. The exact protein function has not yet been determined. At least 65 patients with different genetically confirmed* TMEM70* mutations were reported to date [9], many from consanguineous parents of Roma ethnic background [10]. Although genetic data are available for most patients, ultrastructural examination of muscle tissue has rarely been performed in patients with* TMEM70* mutations. In one patient with compound heterozygous mutations (c.117_118dupGT in exon 1 and c.317-2A>G in intron 2) concentric cristae and reduced OXPHOS proteins including F1*α* as assessed by immunogold labeling were described [11]; in another patient loss of cristae was described [12]. Upon transfection of wild type* TMEM70* into patients' fibroblasts mitochondrial morphology was restored [5].

Herein, we provide a detailed pathological analysis of patients with a* TMEM70* defect and report on the successful stabilisation of life-threatening metabolic crises by combined symptomatic and experimental anaplerotic therapy.

## 2. Materials and Methods

### 2.1. Clinical and Epidemiological Data

**Figure 1:**
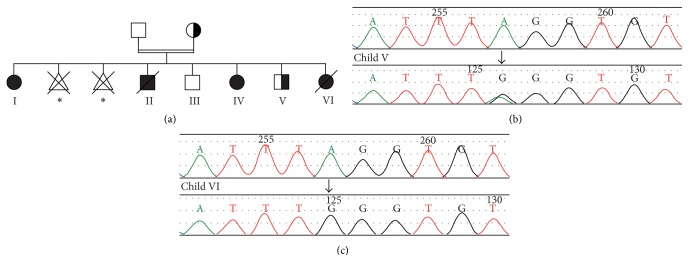
Pedigree of family with* TMEM70* mutation and genetic data. (a) Black symbols indicate affected individuals, half-filled symbols indicate heterozygous individuals, and white symbols indicate phenotypically healthy individuals; circles: female; squares: male; triangle: miscarriage; crossed symbol: patient deceased; double lines: consanguineous marriage. The consanguineous parents had four affected children, two healthy children, and two abortions. The exact time of the miscarriages is not known (star). The half-filled individuals have a confirmed homozygous* TMEM70* c.317-2A>G mutation. Sequencing revealed a heterozygous mutation in the mother and child V ((b), sequencing detail of the heterozygous child V, arrow indicates site of mutation) and a homozygous mutation in children I, IV, and VI ((c), sequencing detail of the homozygous child VI, arrow indicates site of mutation). For the affected and deceased child II and the phenotypically healthy child III no genetic information is available.

A consanguineous couple of Roma background with 2 abortions and 6 living births was examined (Figure 1(a)). During observation time, 2 of 6 children died. A detailed overview about the clinical and epidemiological data is provided in Table 1. Parents gave informed consent for clinical and genetic examination of their children. Standard laboratory parameters were assessed according to routine diagnostic protocols.

### 2.2. Genetic Analyses

Genomic DNA of the affected children and their family members was studied. Analysis for mtDNA deletions was performed by standard methods (Long Range-PCR, Southern Blot analysis). Sanger sequencing of the* TMEM70* gene in children V and VI was performed by standard methods using the following primer set:

 
*TMEM70*-3.1fw_4692 gcactGTATTTATGGTTTGATTTTG.
 
*TMEM70*-3.1rv_4692 ATGCCGTTTCTCTTCACTGG.
 Mutation nomenclature referred to* NM_017866.5*.

### 2.3. Isolation of Mitochondrial Complexes

Isolation of mitochondrial membranes from 15 mg skeletal muscle (wet weight) and solubilization of OXPHOS complexes by dodecylmaltoside were performed as described [13]. Protein complexes were separated by high resolution clear native electrophoresis (hrCNE) [14] on 4 to 13% acrylamide gradient gels. An ATP hydrolysis/lead phosphate precipitation assay was used to detect ATP synthase. Mitochondrial complexes were stained by Coomassie Blue G-250 on the same gel. For separation of individual subunits of protein complexes, native lanes were separated by two-dimensional sodium dodecyl sulfate polyacrylamide gel electrophoresis (2D SDS-PAGE) [15] and stained with silver.

### 2.4. Histology and Enzyme Histochemistry

Muscle tissue was obtained by skeletal muscle biopsies. Snap-frozen tissues were stored at −20°C until use. The specimens were cut at 7 *μ*m thick slices using a manual cryostat system (Leica CM 1900, Wetzlar, Germany) and were stained according to standard protocols including standard myopathological and enzyme histological stains and finally mounted in Entellan (Merck Millipore, Darmstadt, Germany). Pathological analysis of the samples was performed by at least two experienced neuropathologists (DST, MM) using light microscopy (Olympus BX41, Hamburg, Germany). Due to sending the material to reference centers, not all slides could be retrieved from our archive.

### 2.5. Electron Microscopy

Transmission electron microscopy (TEM) was used to visualize ultrastructural changes in skeletal muscle tissue. Therefore, muscle tissues were initially fixed overnight using 2.5% glutaraldehyde buffered in cacodylate. The embedding procedure comprised fixation in 1% osmium tetroxide, dehydration in a graded ethanol series with an incubation step with uranyl acetate (between the 50% and 90% ethanol step), and finally rinsing in propylene oxide. The specimens were then embedded in epoxy resins that polymerized for 16 h at 60°C. After embedding, semithin sections (0.5 *μ*m) were cut using an ultra-microtome (Leica Ultracut UCT, Deerfield, IL, USA) with a diamond knife. Sections were stained with toluidine blue, placed on glass slides, and examined by light microscopy to select appropriate areas for ultrathin preparation. Ultrathin sections (50–70 nm) were cut again using an ultra-microtome. Sections were mounted on copper grids and contrasted with uranyl acetate for 2-3 h at 42°C followed by lead citrate for 20 min at room temperature. Imaging was performed using a FEI Tecnai G2 Spirit Biotwin TEM (Hillsboro, OR, USA) at an operating voltage of 120 kV. Interpretation of the results was carried out by two experienced neuropathologists (DST, MM).

## 3. Results

**Figure 2:**
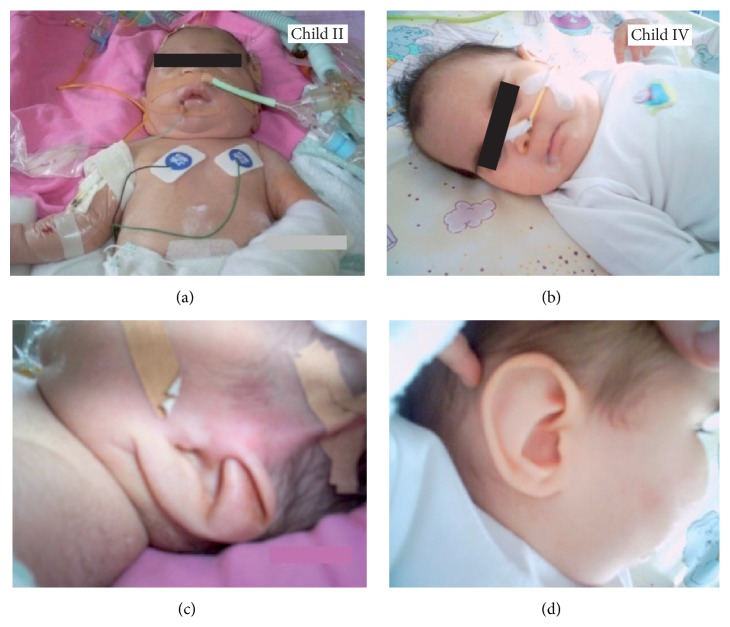
Macroscopic dysmorphic signs in familial* TMEM70* mutation. (a, c): child II, one day after birth; (b, d): child IV, at the age of 11 months. Both patients present with prominent dysmorphic ears.

A family with consanguineous Roma pedigree had 4 clinically affected children, two abortions in the third month of pregnancy, and two children who were reported to be healthy (Figure 1). The affected children initially showed 3-MGA with lactate acidosis, left ventricular hypertrophic cardiomyopathy, and hepatomegaly. The children presented with failure to thrive and displayed morphological stigmata including hypertrophic deeply seated ear cartilage and a bird's face (Figure 2). Two of the children (children II and VI) died of cardiac failure and therapy refractory lactic acidosis within three days after birth. Two affected children (children I and IV) are long-term survivors with a follow-up of 13 and 7 years. Both children showed symptoms of heart failure due to hypertrophic cardiomyopathy and suffered from recurrent metabolic crises usually triggered by respiratory or other infections since birth. Both had significant psychomotor delays and developed intellectual disability necessitating special school enrolment. Among several differential diagnoses an impaired OXPHOS system due to a mitochondrial disease was suspected.

Since birth, the children suffered from life-threatening metabolic crises, which were most severe during the postnatal period but reoccurred later upon situations with elevated energy demand, for example, infections. In those metabolic crises the children presented with sucking weakness, vomiting, diarrhea, irritability, and in more advanced stages somnolence and hyperventilation caused by a severe, combined lactic and ketoacidosis. The emergency treatment aimed to convert catabolism into anabolism and to compensate acidosis. Because of the limitations of glucose as energy supplement in patients with OXPHOS defects, which would result in an even more severe acidosis, alternative energy sources such as amino acids and possibly lipids were administered. In our patients, the following i.v. regimen was repeatedly applied with success: glucose (max. 7 g/kg body weight/day), amino acids (2 g/kg body weight/day), and lipids (2 g/kg body weight/day). In refractory situations, a combination of cofactors and essential amino acids was given including sodium citrate, glutamine, sodium succinate, sodium aspartate,* L*-carnitine, coenzyme Q10, and vitamin C (Table 2). Since the age of six months, child I was supposed to be on treatment with cofactors to improve cell energy transfer consisting vitamin E, coenzyme Q10, vitamin C, lipoic acid, niacin amide, and* L*-carnitine, but because of poor compliance child I may not have received the treatment most of the time.

**Figure 3:**
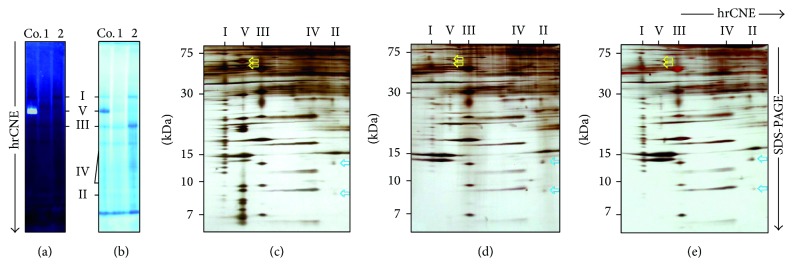
2D BN-PAGE reveals complex V deficiency. Analysis of muscle tissue in native gels revealing an almost complete deficiency of the mitochondrial ATP synthase (complex V) (a, b). I-V, mitochondrial complexes I-V; Co., control; 1, child I; 2, child II. (a) Mitochondrial complexes from homogenized skeletal muscle biopsies were solubilized by dodecylmaltoside, separated by hrCNE, and analyzed by ATP hydrolysis/lead phosphate precipitation assay. (b) Gel A was restained with Coomassie to display other complexes of the respiratory chain. Separation of the subunits of mitochondrial complexes by second-dimension SDS-PAGE showed drastically reduced but fully assembled ATP synthase (complex V) in the patients (c–e). I-V, mitochondrial complexes I-V. Mitochondrial complexes from patient skeletal muscle were solubilized by dodecylmaltoside and separated by hrCNE. The strip of the native gel was used for 2D SDS-PAGE to resolve the subunit composition of the mitochondrial complexes, as shown in silver stained gels for (c): control, (D): child I, and (e): child II. Yellow arrows mark *α* and *β* subunits of ATP synthase. Blue arrows indicate the SDHC and SDHD subunits of complex II.

Biochemical analysis of mitochondrial complexes of skeletal muscle biopsies from patients II and IV by high resolution clear native electrophoresis (hrCNE) in-gel ATP hydrolysis/lead phosphate precipitation assay and Coomassie stain detected an almost complete ATP synthase deficiency (Figures 3(a)-3(b)). 2D hrCNE/SDS-gels revealed again drastically reduced amounts of fully assembled ATP synthase (Figures 3(c)–3(e)). Sanger sequencing of the mtDNA did not detect any causative mutation. Based on the predominant cardiac symptoms mutations in a COX assembly factor* SCO2* were excluded in child I. In child IV mtDNA deletions and the common mutation m.8993 in* MT-ATP6* were excluded. As complex V deficiency in Roma families had been linked to* TMEM70*, initially described by Spiegel et al. [10], Sanger sequencing of* TMEM70* detected the previously reported mutation c.317-2A>G in a homozygous form in the 3 affected children (children I, IV, and VI) (Figures 1(b)-1(c)). The mother and the healthy 5th child were heterozygous. The father was not willing to be tested, but in this family a paternal heterozygous mutation of* TMEM70* can be assumed.

**Figure 4:**
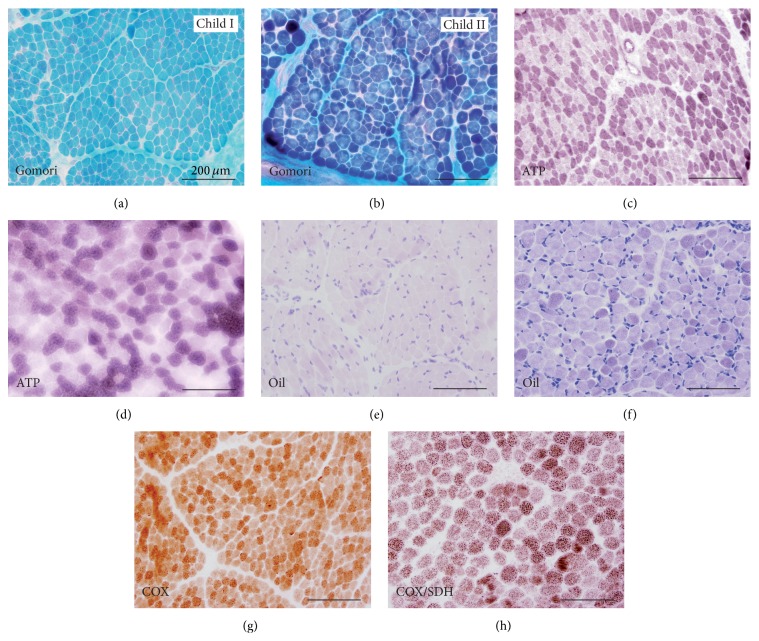
Light microscopy of patients with* TMEM70* mutations. (a, c, e, g): child I, Gomori, ATPase, Oil Red O, and COX; (b, d, f, h): child II, Gomori, ATPase, Oil Red O, and COX/SDH. Muscle biopsies were snap-frozen and stained according to routine protocols. Apart from varying fibre size in child II, muscle tissues appear mainly unremarkable. The specimens especially lack typical histological hallmarks of mitochondriopathies such as ragged-red fibres or COX-deficient fibres.

**Figure 5:**
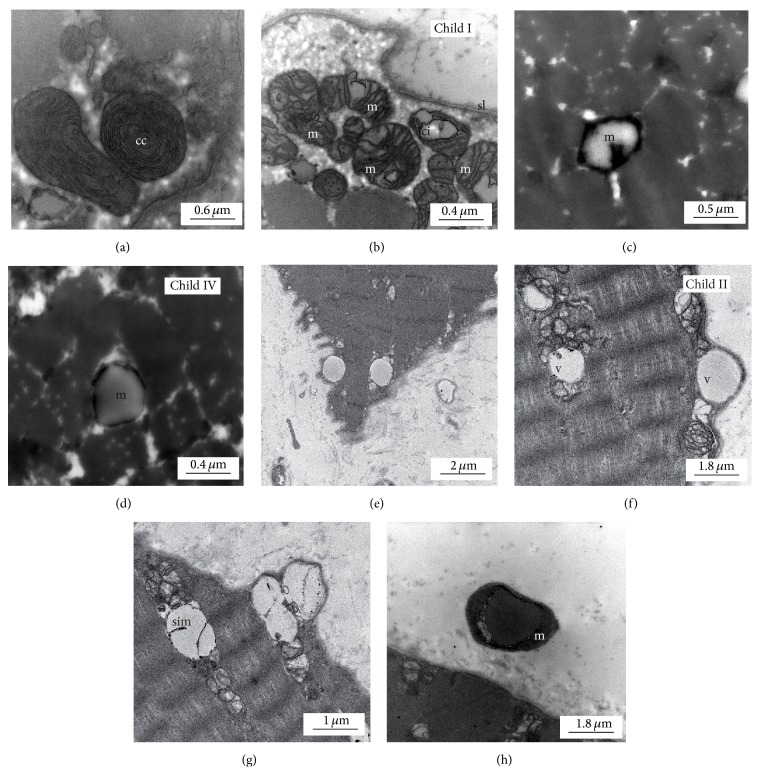
Electron microscopy reveals altered mitochondria morphology. Ultrastructural findings in muscle biopsies, child I (a, b), child IV (c, d), and child II (e–h), magnification as indicated by the scale bar. We detected abnormal mitochondria with concentric cristae (a, cc) as well as subsarcolemmal (sl) accumulation of swollen mitochondria (m) with crystalline inclusions (ci) in child I (b). In child IV, sample quality was not optimal; degenerated mitochondria (m) were detected (c, d). In child II (e–h), we detected accumulations of swollen mitochondria with abnormal cristae structure and separated inner (sim) and outer membrane with vacuoles (v), (g) possibly by intramitochondrial lipid accumulation with cristae aggregation. Additionally, we saw protruding membranes filled with amorphous and electron dense material, most possibly mitochondria (f, g). Extracellular abnormal membranous structures (m, dense globular inclusion) were detached from the main myofibre (h).

Light microscopic analysis of standard hematoxylin and eosin (H and E) stainings and muscle specific enzyme histochemical stainings (children I, II; IV: data not shown) showed apart from varying fibres sizes in child II largely unremarkable myofibres without any specific pathological findings, especially no ragged-red or cytochrome C oxidase- (COX-) negative fibres as a hint for a mitochondrial disease (Figure 4). In contrast, electron microscopy revealed distinct mitochondrial alterations. In the muscle biopsy of child I at the age of one year giant mitochondria with abnormal, sometimes concentric cristae as well as subsarcolemmal accumulation of swollen mitochondria were present (Figures 5(a)-5(b)). Several mitochondria revealed electron dense (possibly cristae fragments) and globular inclusions. In child IV a muscle biopsy was taken on the day of birth, but despite suboptimal sample quality, electron dense material within mitochondria and intramitochondrial lipid accumulation with cristae aggregation were detectable (Figures 5(c)-5(d)). Electron microscopy of child II muscle biopsy taken at the age of two days showed accumulation of swollen mitochondria with abnormal cristae structure as seen in child I but with an additional separation of the inner and outer membranes and vacuolation possibly resulting from cristae aggregation (Figures 5(e)–5(h)). Again subsarcolemmal accumulations of mitochondria with crystalloid inclusions were present. Protruding vacuole-like amorphous and electron dense mitochondrial material from the muscle fibre invoked the term “exocytosis of mitochondria.” This observation was strengthened by the finding of extracellular, membranous structures with dense globular inclusions which were detached from the corresponding myofibre (Figure 5(h)).

## 4. Discussion

The clinical findings together with lactic acidosis and 3-MGA accumulation during recurrent metabolic crises in the affected children of the observed family were highly suggestive of mitochondrial disease [16]. Biochemical analysis in muscle revealed a reduced or abolished complex V assembly that was caused by the absence of the protein complex (Figure 3). Meanwhile, the candidate gene* TMEM70* causing a complex V deficiency with morphological stigmata, cardiomyopathy, and metabolic crises frequently occurring in families of Roma ethnic background had been described [8]. The homozygous* TMEM70* (c.317-2A>G) mutation in intron 2 was found in children I, IV, and VI and segregated with the disease within the family. This mutation has been shown to result in aberrant splicing and a loss of the* TMEM70* transcript [8]. To date, at least 14 additional* TMEM70* mutations have been described leading to a broad clinical spectrum such as hypospadia [10] or pulmonary arterial hypertension [17], as summarized in Magner et al. [18].

Two of the four affected children died within 60 hours postpartum of metabolic crises and heart failure due to cardiomyopathy refractory to treatment. In all children, the initial treatment of metabolic crises aimed to stop catabolism and establish an anabolic metabolism. Therefore energy supply avoiding an increase of lactate through glucose was provided with offering alternative substrates for the Krebs cycle, such as amino acids and fat. In refractory situations, a mix of cofactors and additional essential amino acids were added to relieve the metabolic crisis. The concept of supplementing essential precursors of the Krebs cycle is referred to as anaplerotic therapy [19]. An effect of* L*-carnitine, an important compound inside the therapy regimen, was shown in a liver disease mouse model, where mitochondrial abnormalities improved upon treatment with* L*-carnitine [20]. Standardized clinical trials evaluating anaplerotic therapy are lacking [21], but out of different treatment strategies in 48 patients with* TMEM70* deficiency some general recommendations have been deduced by Magner et al. consisting basically of moderate glucose supplementation, high dose lipid infusions, and acidosis correction via bicarbonate infusions [18]. The effect of *L*-carnitine, coenzyme Q10, and vitamin C administered here cannot be evaluated exactly; still, given the few therapeutic options in mitochondrial diseases, these supplements additionally improved the situation during metabolic crises in our patients.

In the course of the diagnostic process, muscle biopsies were taken from three affected children. Light microscopy analyses did not reveal any major pathological findings and were especially lacking ragged-red or COX-deficient fibres that would have been suggestive of a mitochondrial disease (Figure 3). Interestingly, muscle biopsy from a non-Roma patient with compound heterozygous* TMEM70* mutations (c.317-2A>G and c.494G>A, p.Gly165Asp) taken at 30 months of age demonstrated the presence of ragged-red fibres, which were not present in our patients nor in the other reported cases with different mutations [22, 23]. It is not clear by which effects the presence of ragged-red fibres can be explained in the relatively mildly affected child above.

Additional electron microscopic investigations in our patients' muscle tissues (Figure 5) revealed a spectrum from mild to more severe pathological alterations including giant mitochondria, subsarcolemmal accumulation of mitochondria, globular inclusions, concentric cristae formation, cristae fragmentation, and crystalloid inclusions. Child IV showed most extended morphologic alterations of muscle tissue and mitochondria and died within 60 hours after birth. Children I and IV with milder ultrastructural alterations are long-term survivors. Prognostic impact of the ultrastructural findings remains speculative.

Jonckheere et al. reported a fragmented mitochondrial network and swollen, irregularly shaped mitochondria with partial to complete loss of the cristae in fibroblasts of a patient with a deletion of* TMEM70* exon 2 (g.2436–3789). The successful restoration of mitochondrial morphology by supplement of the wild type* TMEM70* vector in patients' fibroblasts directly links the observed abnormalities to the defect [5]. In summary, there is much evidence that the complex V defect resulting from the* TMEM70* mutations directly affects mitochondrial morphology.

Our electron microscopic examination of the muscle biopsies also showed protrusion of mitochondrial material at the myofibres' surfaces as well as circular extracellular membranous structures in close vicinity of the myofibres (Figure 5). Paracrystalline inclusions can be frequently seen in mitochondrial disorders [24], but perisarcolemmal accumulated mitochondria have not been reported so far in complex V defects. To date, only single reports about the so-called “free-floating” mitochondria exist. In* Sod1* mutant mice modelling amyotrophic lateral sclerosis (ALS), extracellular free-floating mitochondria have been described in the brainstem. They were located extracellularly between capillaries and astrocyte processes and accompanied by edema. In this case the “free-floating mitochondria” were interpreted as a result of a blood brain barrier breakdown related to a basement membrane defect in* Sod1* mutant mice [25]. Cells are believed to have acquired mitochondria from invading aerobic bacteria more than a billion years ago employing ATP as energy source and accepting the bacteria's residency inside the protoeukaryotic cell. This model is referred to as the “endosymbiotic hypothesis” [26, 27]. In mitochondriopathies aggregates of degenerated mitochondria can be observed frequently subsarcolemmal. Possibly the contractile apparatus is less affected if the mitochondria degradation takes place in a subsarcolemmal position. As a highly speculative guess, the process of “exocytosis of mitochondria” could be a way of eliminating mitochondria in a state of extensive mitochondrial damage as an alternative disposal mechanism. This might be a reverse step to phylogenetic uptake of mitochondria according to the endosymbiotic theory. In yeast, the phenomenon of mitochondrial regression is connected to the petit phenotype and can be experimentally induced, which is then called rho status [28, 29]. Physiologically, the mitochondrial turnover takes place via mitophagy, the specific autophagic elimination of mitochondria. If mitophagy is blocked experimentally, this leads to increased reactive oxygen species (ROS) production and senescence-like morphology chances including concentric cristae formation [30, 31]. Important genes related to mitophagy are* PINK1* and* PARKIN* [32], both known to be mutated in familial forms of Parkinson's disease. In* PINK1 *−/− mice changes in mitochondria size, but not in the overall morphology, were seen ultrastructurally [33]. Still, the phenomenon of mitochondrial exocytosis might be a cleaning mechanism of defect mitochondria; however, further studies are necessary to decipher the relevance of these observations.

## 5. Conclusion

In summary, we report on 4 children with* TMEM70* defect from a consanguineous Roma family resulting in complex V deficiency, 3-MGA, and particular ultrastructural mitochondrial alterations. While facing metabolic crises, anaplerotic therapy was administered attenuating clinical symptoms. Although clinical studies for anaplerotic therapy in patients with* TMEM70* mutations are lacking, we propose anaplerotic therapy for affected children during metabolic crises as a promising treatment approach. Our oldest patient with now 14 years of age indicates that long-term survival can be achieved in* TMEM70* patients. This might be important in genetic counselling of parents with affected children.

## Figures and Tables

**Table 1:** Clinical and epidemiological data.

	Child I, ^*∗*^2000	Child II, ^*∗*^2004	Child III, ^*∗*^2005	Child IV, ^*∗*^2006	Child V, ^*∗*^2010	Child VI, ^*∗*^2013
Pregnancy and birth details	Intrauterine reduced child movements, APGAR 7/3/5, cord pH 7.17	Anhydramnion, APGAR 5/7/7, cord pH 7.21	n.a.	Anhydramnion, APGAR 7/8/7, cord pH 7.16	n.a.	Reduced child movements, anhydramnion since the 29th week of gestation, APGAR 6/6/n.a., cord pH 7.29

Morphological stigmata	Dysmorphic ears	Dysmorphic ears	n.a.	Dysmorphic ears	n.a.	Dysmorphic child

Organ impairment	Noncompaction cardiomyopathy (NCCM), hepatomegaly	Noncompaction cardiomyopathy (NCCM), hepatomegaly	n.a.	Noncompaction cardiomyopathy (NCCM), hepatomegaly	n.a.	Noncompaction cardiomyopathy (NCCM), hepatomegaly,

Clinical course	Failure to thrive, mild retardation, rarely metabolic crises	Death 55 hours postpartum	No hospitalisation	Failure to thrive, retardation, isolated metabolic crises	n.a.	Death 58 hours postpartum

Long-term course	Mental retardation, reduced growth, clinical contact during respiratory infections, isolated metabolic crises; child was followed up to the age of 13 years		Reported to be healthy	Mental retardation, reduced growth, clinical contact during respiratory infections, isolated metabolic crises, periodic nutrition via endogastric tube; child was followed up until age of 7 years	Reported to be healthy	

Routine laboratory	Lactate 15 mmol/L, 3-MGA elevation	Lactate 32 mmol/L, 3-MGA elevation	n.a.	Lactate 17 mmol/L, 3-MGA elevation	n.a.	Lactate 39 mmol/L, 3-MGA not tested

Muscle light microscopy	No path. findings, no COX-negative fibres, no ragged-red fibres	No path. findings, no COX-negative fibres, no ragged-red fibres	n.a.	No path. findings, no COX-negative fibres, no ragged-red fibres	n.a.	No muscle biopsy performed

Muscle biochemistry	Reduced activity in complexes I, II, III, and IV, complex V-activity 69 nmol/min/mg	Almost complete deficiency on native-PAGE	n.a.	Reduced activity in complex I, almost complete deficiency on native-PAGE	n.a.	n.a.

Genetic findings	No changes in SCO2 (cytochrome c oxidase assembly gene)	n.a.	n.a.	No mtDNA deletions or m.8993 mutation in MT-ATP6 gene (8993 restriction fragment length polymorphism)	n.a.	n.a.

Status of TMEM70 c.317-2A-G	Homozygous	n.a.	n.a.	Homozygous	Heterozygous	Homozygous

^*∗*^Year of birth; for example, ^*∗*^2000 means the child was born in 2000.

**Table 2:** Anaplerotic therapy.

Substance	Individual dose and frequency	Application
Sodium citrate 3.13%	3 × 500 mg × d (*∗*)	p.o. (i.v.)
Glutamine	3 × 500 mg × d	i.v.
Sodium succinate	3 × 100 mg × d	i.v.
Sodium aspartate	3 × 100 mg × d	i.v.
*L*-Carnitine	3 × 100 mg/kg body weight × d	p.o.
Coenzyme Q10	3 × 100 mg/kg body weight × d	p.o.
Vitamin C	1 × 10 mg/kg body weight × d	p.o.

(*∗*): dosage control via urine pH; target urine pH should be above pH = 7.
